# Dual extraction of mRNA and lipids from a single biological sample

**DOI:** 10.1038/s41598-018-25332-9

**Published:** 2018-05-04

**Authors:** Normand Podechard, Simon Ducheix, Arnaud Polizzi, Frédéric Lasserre, Alexandra Montagner, Vincent Legagneux, Edwin Fouché, Fabrice Saez, Jean-Marc Lobaccaro, Laila Lakhal, Sandrine Ellero-Simatos, Pascal. G. Martin, Nicolas Loiseau, Justine Bertrand-Michel, Hervé Guillou

**Affiliations:** 1 INRA UMR1331, ToxAlim, University of Toulouse, Toulouse, France; 2 Irset - Inserm UMR 1085, UFR des Sciences Pharmaceutiques et Biologiques, 35043 Rennes Cédex, France / Biosit SFR UMS CNRS 3480, Université de Rennes 1, 35043 Rennes Cédex, France; 3 Institut National de la Santé et de la Recherche Médicale (INSERM), UMR1048, Institute of Metabolic and Cardiovascular Diseases, Toulouse, France; 4 Université Clermont Auvergne, GReD, CNRS UMR 6293, INSERM U1103, 28, place Henri Dunant, BP38, F63001 Clermont-Ferrand, France; 5 Centre de Recherche en Nutrition Humaine d’Auvergne, 58 Boulevard Montalembert, F-63009 Clermont-Ferrand, France; 6 Metatoul-Lipidomic Facility, MetaboHUB, Institut National de la Santé et de la Recherche Médicale (INSERM), UMR1048, Institute of Metabolic and Cardiovascular Diseases, Toulouse, France

## Abstract

The extraction of RNA and lipids from a large number of biological samples is time-consuming and costly with steps required for both transcriptomic and lipidomic approaches. Most protocols rely on independent extraction of nucleic acids and lipids from a single sample, thereby increasing the need for biological material and inducing variability in data analysis. We investigated whether it is possible to use a standard RNA extraction procedure to analyze not only RNA levels, but also lipids in a single liver sample. We show that the organic phase obtained when using standard reagents for RNA extraction can be used to analyze lipids, including neutral lipids and fatty acids, by gas chromatography. We applied this technique to an analysis of lipids and the associated gene expression pattern in mice with hepatic steatosis induced by pharmacological activation of nuclear receptor LXR.

## Introduction

Both transcriptomic^1^ and lipidomic^2^ analyses have become accessible and essential tools for investigators in the field of lipid research. However, most lipid analysis and gene expression assays involve independent, costly, and time-consuming extractions.

Given that most RNA extractions are based on a phase split between chloroform and phenol, we thought it would be worth evaluating whether the organic phase can be used for complementary lipid analysis. We tested a protocol adapted from Bligh and Dyer’s extraction^3^ and applied it to the remaining organic phase after the aqueous phase had been used for RNA extraction. We used liver tissue and hepatic cell lines to assess the method we developed and validated the protocol by analyzing samples from wild-type mice and transgenic mice lacking liver X receptor (LXR) α and β (NR1H3 and NR1H2, respectively)^4^, two transcription factors known to play a central role in cholesterol and fatty acid metabolism^5^. By administering T0901317, a pharmacological agonist of both LXRs^6^, we induced hepatic steatosis in wild-type mice but not LXR^−/−^ mice^4,6^. This steatosis occurs through an important increase in the expression of genes involved in fatty acid synthesis^6^, also called *de novo* lipogenesis. In this experimental setting, we tested whether LXR-induced steatosis correlates with changes in the expression of major lipid droplet proteins from the CIDE, PLIN, and FITM families^7^. In addition, sample-specific networks^8^ were analyzed, indicating *Dgat*2 as an atypical LXR-sensitive gene involved in triglyceride synthesis^9,10^ and down-regulated by pharmacological activation of LXR.

## Results

### Comparison of RNA extraction protocols

**Figure 1:**
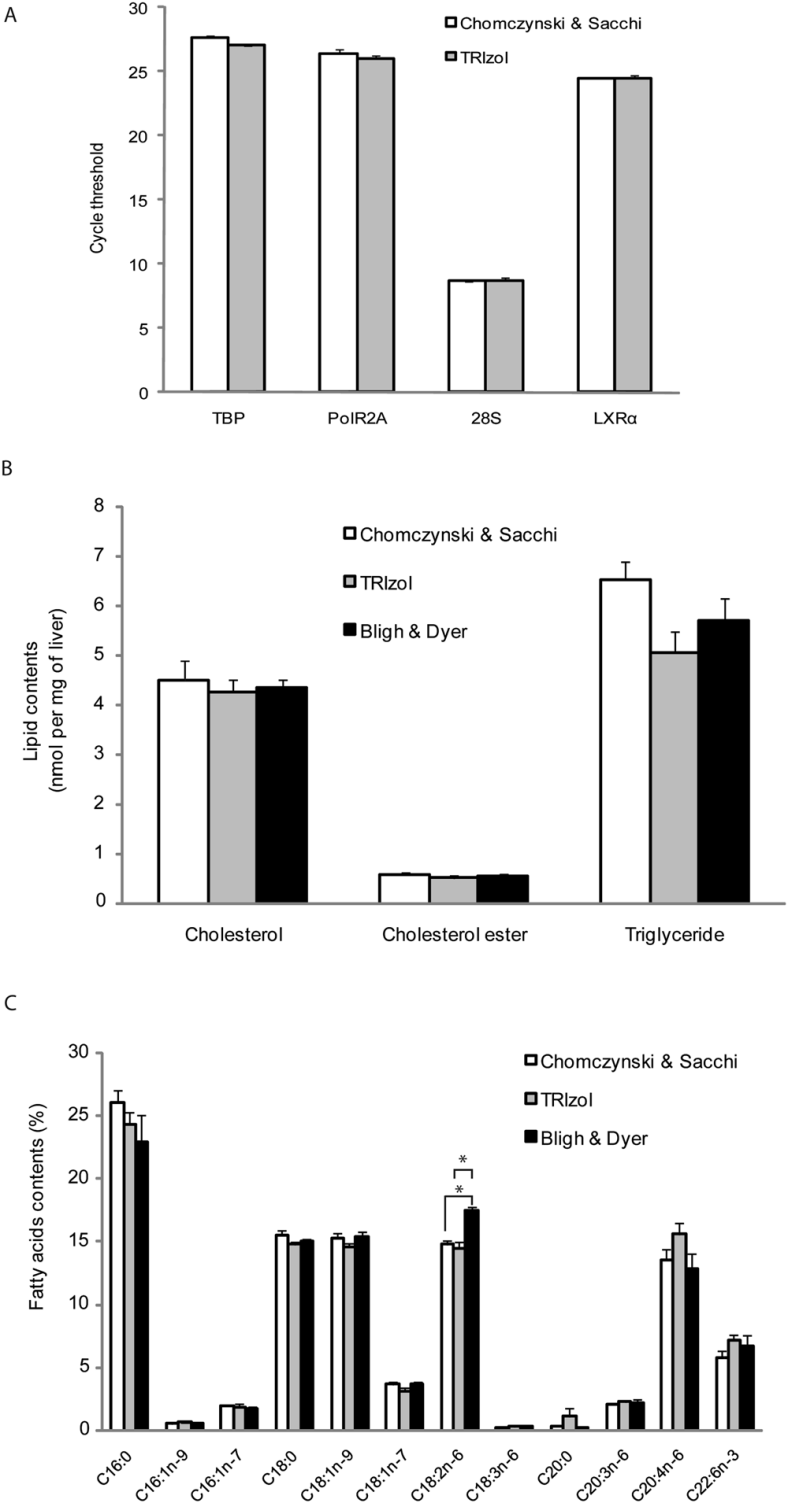
Commercially available Trizol can be used to extract mRNA, total lipids, and total fatty acids from mouse liver samples. (**A**) Commercially available Trizol was compared to the Chomczynski & Sacchi method for mRNA extraction and (**B**,**C**) to Bligh & Dyer for lipid extraction. RNAs were analyzed by qPCR and lipids by gas chromatography. Data are presented as means ± s.e.m. *P < 0.05 compared to Bligh and Dyer, t-test, n = 4 samples per group.

First, we compared the two RNA extraction protocols by measuring the level of various RNAs when prepared from the same liver samples. As shown in Fig. 1A, the RNA levels were similar regardless of the method. The two protocols, one relying on a commercially available reagent (TRIzol) and the other on a well-established method^11^, were further used to investigate whether lipids could be analyzed from the remaining organic phase.

### Comparison of lipid profiles after RNA extraction

Next, we measured abundant neutral lipids, specifically triglycerides, cholesterol, and cholesterol esters, in total liver lipid extracts using different protocols (Fig. 1B). We compared the amount of these lipids to those obtained after standard lipid extraction and found no significant difference. Thus, lipid can be extracted and quantified from the remaining organic phase after RNA extraction. Consistent with this first finding, we also found that such extraction can be used for estimating total fatty acids (Fig. 1C). Interestingly, we found that, depending on the method used, the relative abundance of C18:2n-6 may be underestimated. We concluded that both commercially available reagents and standard methods can be used for dual extraction of mRNA and lipids from a single sample. To further investigate this, we performed several experiments with the commercially available reagent.

### Estimation of the organic phase volume

We estimated the volume of the organic phase using radiolabeled [1-^14^C] palmitic acid. We checked that, under conditions of mRNA extraction with TRIzol, the upper phase contained less than 1% of the total radioactivity (Supplementary Table 1). We could also determine the organic phase volume from the amount of radioactivity contained in 25 µL carefully extracted from the organic phase, estimating that the organic phase was approximately 560 µL (volumes used: TRIzol 1 mL, Chloroform 200 µL, data not shown). This tip is interesting in order to avoid trying to extract all of the remaining organic phase for lipid quantification.

### Linearity of RNA and lipid assays after dual extraction from a single sample

**Figure 2:**
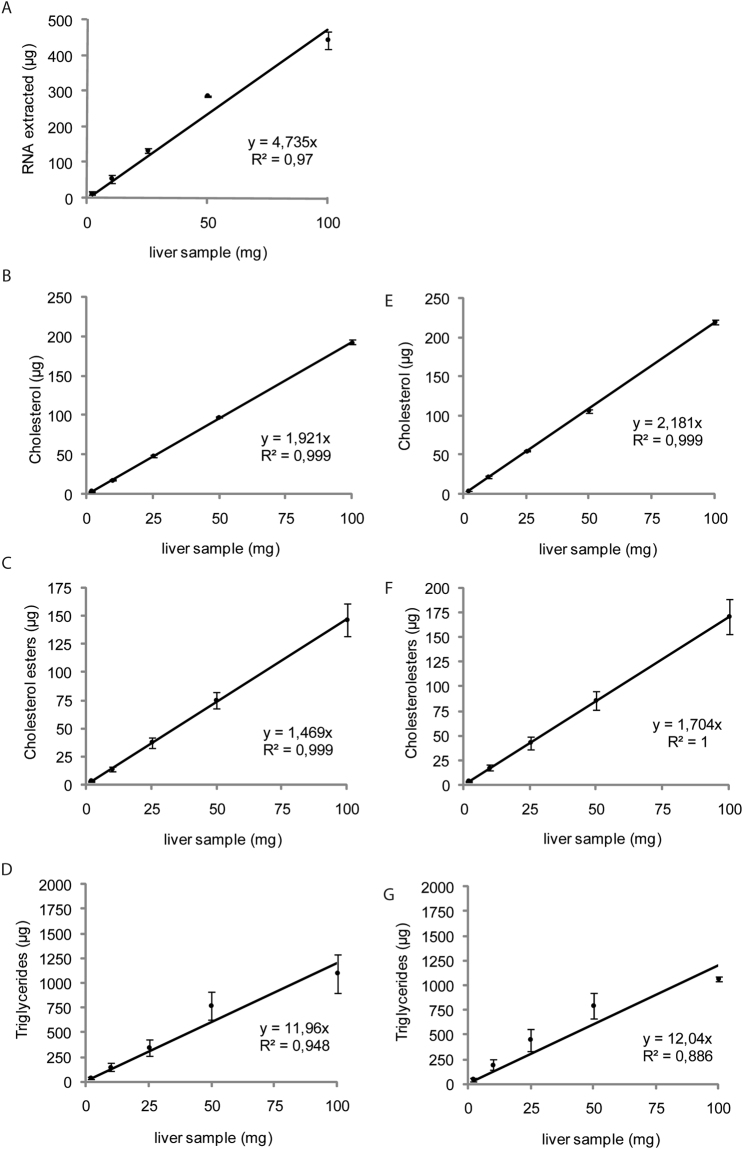
Trizol can be used to extract total lipids from mouse liver samples over a wide range of weights. (**A**) Commercially available Trizol was used to extract mRNA and (**B**) cholesterol, (**C**) cholesterol esters, and (**D**) triglycerides from mouse liver samples of 1 to 100 mg. (**E**) Bligh & Dyer was also used for cholesterol, (**F**) cholesterol ester, and (**G**) triglyceride extraction from mouse liver samples of 1 to 100 mg. RNAs were analyzed by qPCR and lipids by gas chromatography. Data are presented as means ± s.e.m. (n = 4 samples per group).

**Figure 3:**
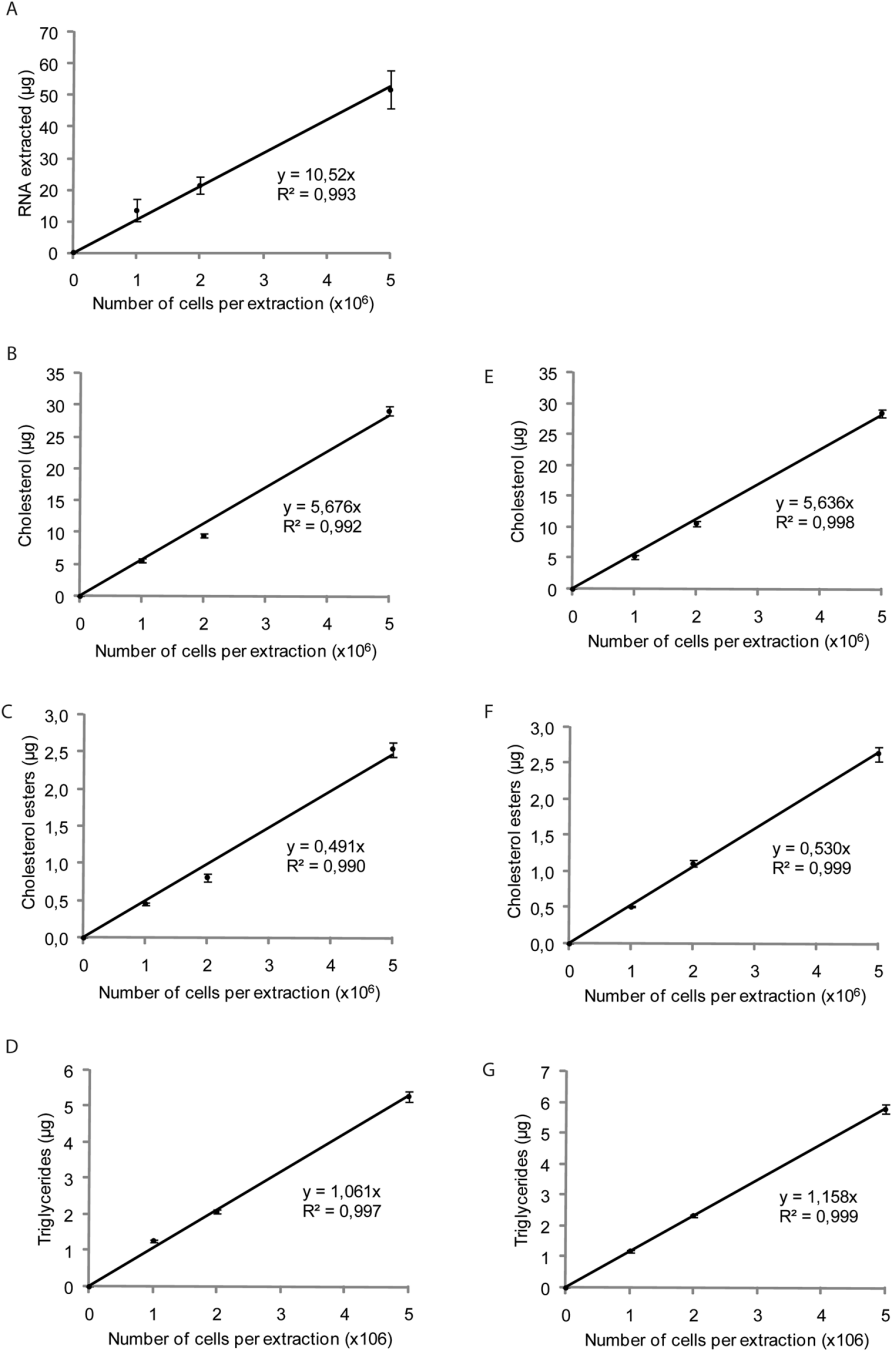
Trizol can be used to extract total lipids from hepatocytes. (**A**) Commercially available Trizol was used to extract mRNA and (**B**) cholesterol, (**C**) cholesterol esters, and (**D**) triglycerides from 1 to 5 × 10^6^ JWZ cells. (**E**) Bligh & Dyer was also used for cholesterol, (**F**) cholesterol ester, and (**G**) triglyceride extraction from 1 to 5 × 10^6^ JWZ cells. RNAs were analyzed by qPCR and lipids by gas chromatography. Data are presented as means ± s.e.m. (n = 4 samples per group).

We then focused on the RNA extraction protocol with the widely used TRIzol reagent, determining whether the protocol can be used within a range of tissue weights and cell numbers, and whether the subsequent RNA assays and lipid quantification are linear. Again, we measured total RNA and major neutral lipids from both liver samples (Fig. 2) and cultured cells (Fig. 3). Regardless of the method used for lipid extraction (Trizol or Bligh & Dyer), the amounts of the various measured lipids were correlated with the amount of tissue (Fig. 2) or number of cells (Fig. 3).

### Validation of dual extraction results

**Figure 4:**
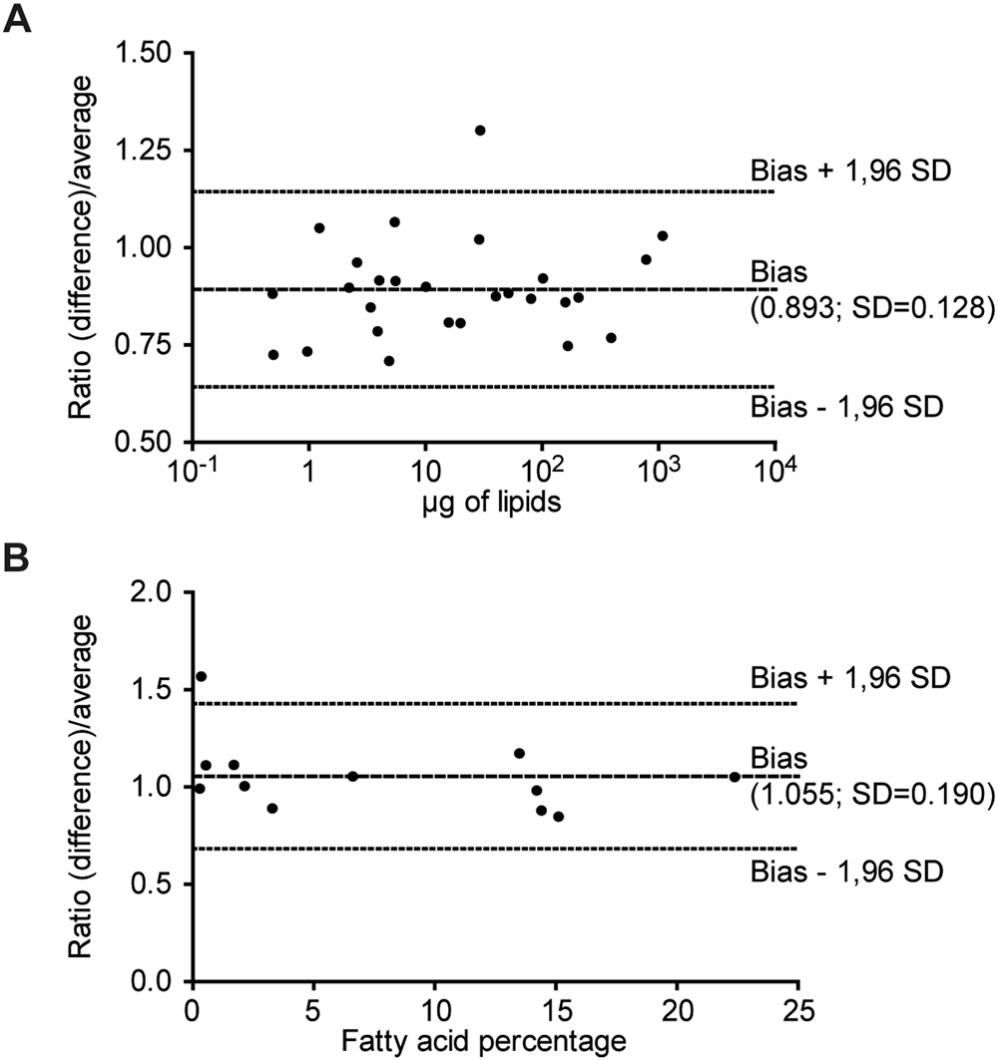
Comparison of methods by Bland-Altman analysis. Bland-Altman plots with mean bias (central line) and 95% limits of agreement (outer lines) for results found after dual extraction and by the reference method (Bligh and Dyer; Figs 2 and 3). (**A**) The analyses were performed for neutral lipid analyses and (**B**) analyses of fatty acid composition. s.d.: standard deviation.

Results obtained after dual extraction from a single sample were compared to those obtained with the classical method of Bligh and Dyer, which was used as a ref.^3^. We analyzed the results using the statistical method of Bland-Altman (Fig. 4). With one exception for each kind of lipid assay, all of the results were included in the 95% limits of agreements (bias ± 1.96 standard deviation) for both neutral lipid analysis (Fig. 4A) and fatty acid composition analysis (Fig. 4B), validating dual extraction from a single sample to quantify lipids.

### Hepatic mRNA and lipids reflecting LXR activity

To test this newly established method in a physiological situation, we focused on the activity of the nuclear LXRs. Pharmacological activation of LXRα and LXRβ triggers marked up-regulation of the hepatic gene expression program involved in *de novo* fatty acid biosynthesis^4,6^. The livers from wild-type (LXR^+/+^) and LXR^−/−^ mice gavaged for 5 days with a potent pharmacological LXR agonist (T0901317) were analyzed for mRNA expression and lipid content.

**Figure 5:**
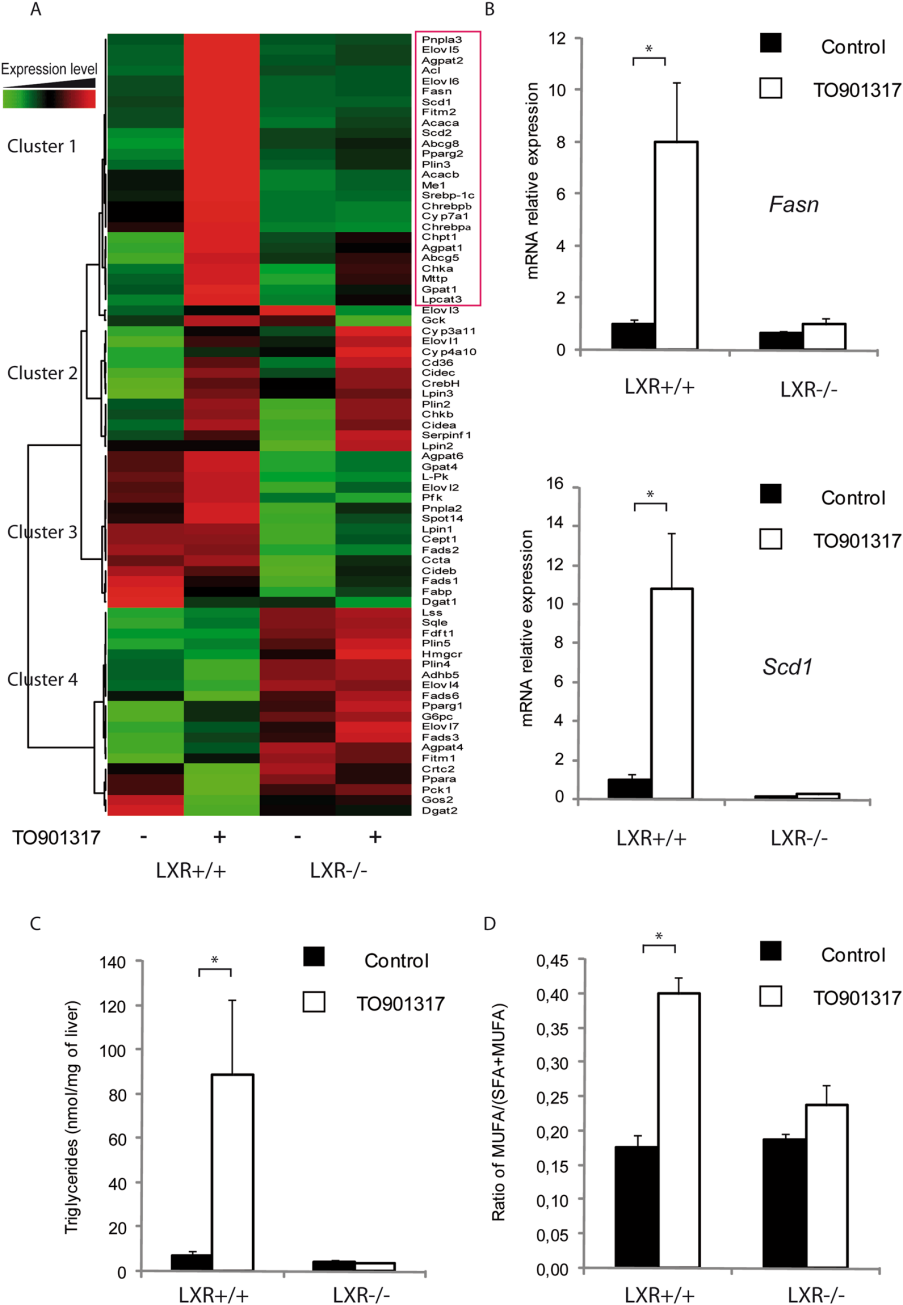
Trizol extraction of both hepatic mRNA and lipids allows detection of changes in gene expression and liver lipids in mice treated with T0901317. (**A**) Hierarchical classification coupled with a heatmap of hepatic gene expression. mRNA levels (74 genes related to lipid metabolism) measured by qPCR. We identified four clusters of genes differentially regulated in response to genotype or LXR activation. (**B**) *Fas* and *Scd1* mRNA quantification by qPCR. (**C**) Liver triglycerides and the ratio of hepatic mono-unsaturated fatty acids (MUFAs) to saturated fatty acids (SFAs) + MUFAs were analyzed by gas chromatography. Data are presented as means ± s.e.m. LXR^+/+^ and LXR^−/−^ mice were treated with vehicle or T0901317 (n = 5 mice per group). *P < 0.05 for the effect of T0901317 on the same genotype.

As expected, we observed a significant change in the expression of genes involved in fatty acid homeostasis (Fig. 5A). The expression of 75 genes related to hepatic lipid metabolism was analyzed by hierarchical clustering. We identified several clusters. Cluster 1 is a large cluster of 26 genes with up-regulated expression in response to T0901317 through a mechanism requiring LXR. This cluster contains well-established LXR target genes, including lipogenic enzymes fatty acid synthase (*Fas*) and stearoyl-CoA desaturase 1 (*Scd*1) (for relative expression see Fig. 5B). In correlation with the increase in lipogenic gene expression, the liver triglyceride level was increased in wild-type mice, but not in mice lacking LXRs (Fig. 5C). Mono-unsaturated fatty acids (MUFAs) were also increased (Fig. 5D), which is consistent with the higher expression of *Scd1* (Fig. 5B). Cluster 1 also contains various other genes involved in fatty acid synthesis, such as *Srebp-1c*, *Chrebp*, *Acaca*, and *Elovl6*, or in fatty acid metabolism, such *Lpcat3* and *Pnpla3*. In addition, this cluster contains genes involved in cholesterol disposal, such as *Abcg5*, *Abcg8*, and *Cyp7α*well-established target genes for LXR. Finally, *Pparγ2*, *Fitm2*, and *Plin3* were also co-localized in this cluster of LXR-sensitive genes.

Three other distinctive patterns of gene expression grouped into other clusters. Cluster 2 contains genes with expression profiles sensitive to T0901317 but do not require LXR. Genes in cluster 3 are up-regulated in wild-type mice compared to LXR^−/−^ mice. In cluster 4, gene expression is up-regulated in LXR^−/−^ mice.

### Single sample network analysis

Because this dual extraction protocol provides information on both the lipid contents and mRNA expression from a single sample, it can be used to investigate the correlation between these biological parameters. In this context, we used data from dual extraction to identify major links between gene expression and lipid homeostasis in the single-sample network (SSN) analysis developed by Liu *et al*.^8^.

**Figure 6:**
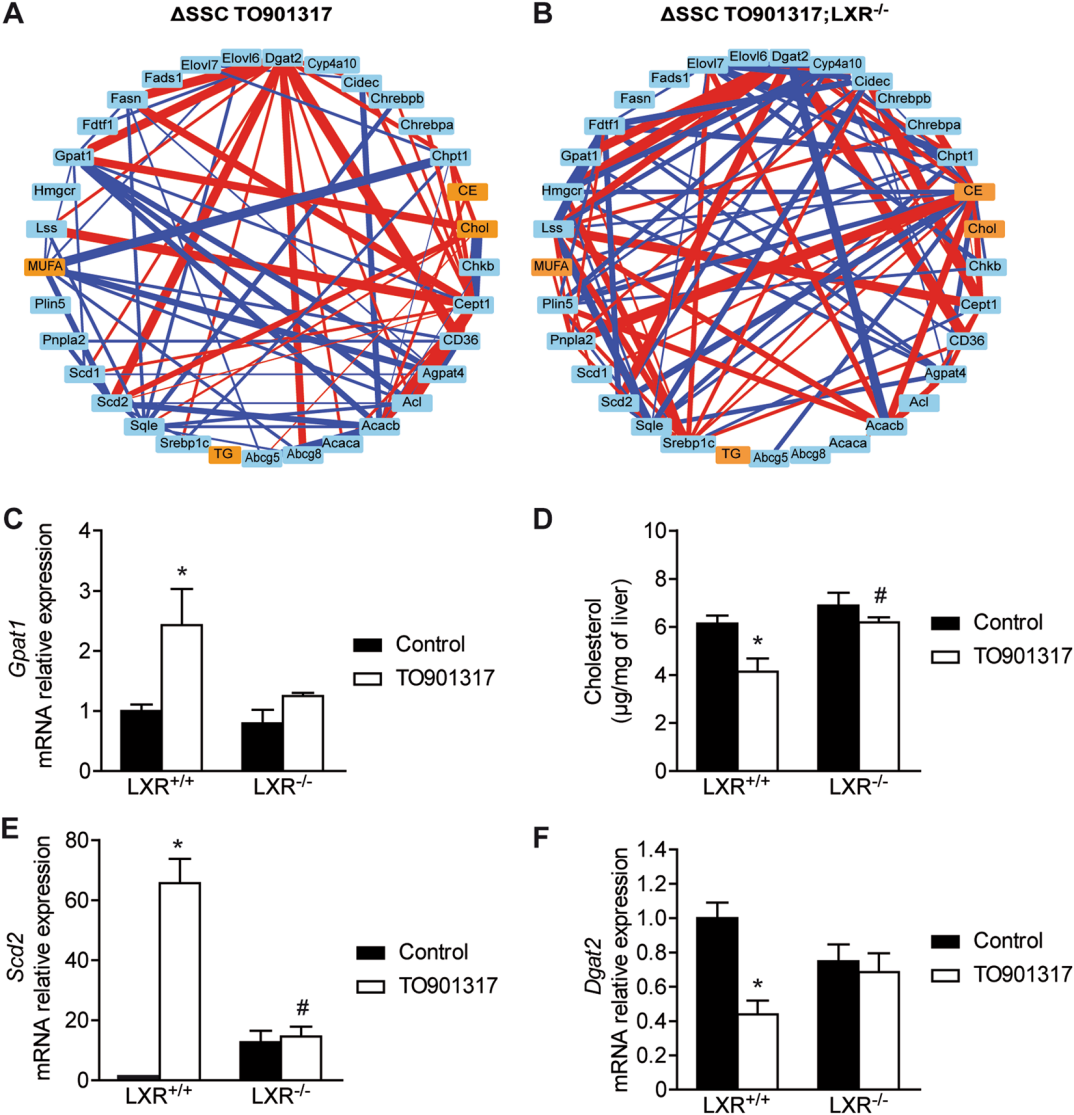
Single-sample network (SSN)-based analyses revealed a potential link between genes. LXR regulation and lipid homeostasis in mice treated with TO901317. (**A**,**B**) SSN-based analysis of the expression of 74 mRNAs (pale blue square) and 4 types of lipids (orange square). CE: cholesterol ester, Chol: cholesterol, MUFA: mono-unsaturated fatty acid, TG: triglyceride. Blue lines represent a gain of correlation, whereas red lines represent a loss of correlation, with a width proportional to the difference in correlation. Only edges of △SSC with P < 0.05 are presented. (**C**) Gpat1 quantification assayed by qPCR. (**D**) Liver cholesterol was analyzed by gas chromatography. (**E**,**F**) Scd2 and Dgat2 mRNA quantification assayed by qPCR. Data are presented as means ± s.e.m. LXR^+/+^ and LXR^−/−^ mice were treated with vehicle or T0901317 (n = 5 mice per group). *P < 0.05 for the effect of T0901317 on the same genotype. ^§^P < 0.05 for the effect of LXR knock-out on the same treatment.

We used the mRNA expression levels from the 74 genes related to lipid metabolism and 4 sets of data from lipid analysis (i.e., cholesterol, cholesterol ester, and triglyceride contents and MUFA/[saturated fatty acid (SFA) + MUFA] ratio) to identify gene expression linked with specific classes of lipids. Significant changes in correlation were found compared to the reference group; all gain or loss of correlation in a pair of biological data could be correlated or anti-correlated. In this way, we could observe major perturbations induced by TO901317 treatment or the absence of LXR (Fig. 6).

Some changes in correlation observed for TO901317-treated mice (Fig. 6A) were not observed in LXR^−/−^ mice treated with TO901317 (Fig. 6B), which suggests a major role of LXR activation in the regulation of the correlated object. For example, a loss of correlation was observed for *Gpat1* (Fig. 6C) and cholesterol (Fig. 6D). In addition, in order to identify a major effect of LXR knock-out, which could be independent of TO901317 treatment, we looked to edges that appear only on ΔSSC TO901317; LXR^−/−^ networks (Fig. 6B). Thus, using these networks, we identified a correlation between the expression profiles of *Scd2* (Fig. 6E) and *Dgat2* (Fig. 6F). Among the genes involved in the biosynthesis of triglycerides, *Dgat2* exhibited an atypical expression profile, as it was the only gene with significantly reduced expression in response to TO901317 (Supplementary Fig. 1).

## Discussion

**Figure 7:**
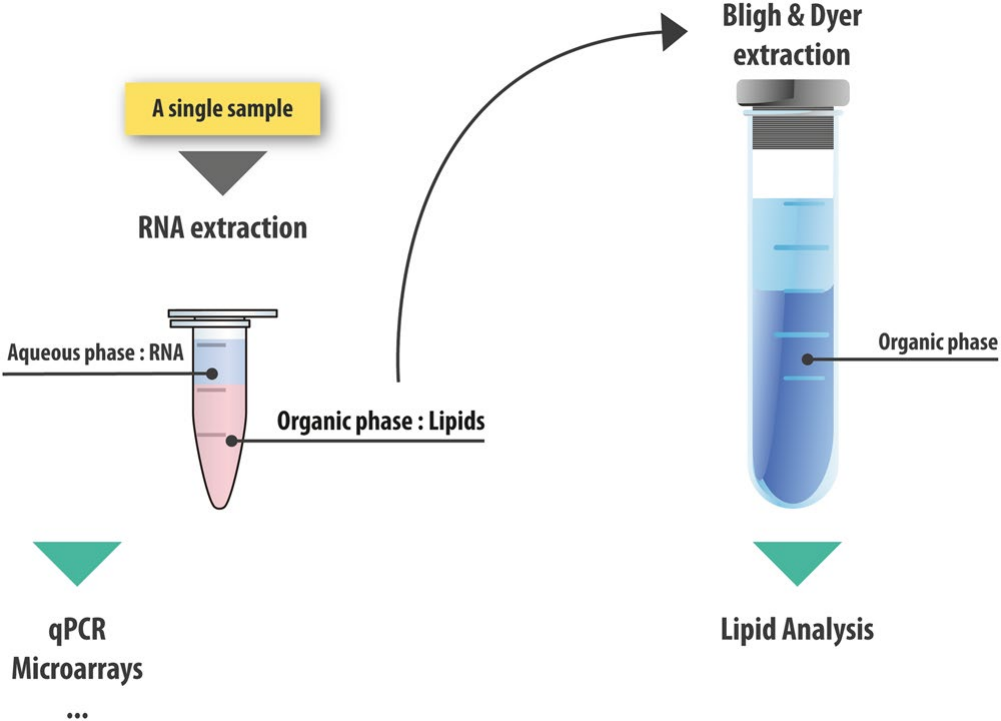
Dual extraction of lipid and mRNA from a single sample.

In this study, we provide evidence that a standard nucleic acid extraction protocol can be adapted to analyze lipids from the same samples (Fig. 7). This protocol is reliable for preparing lipids from both cell culture and tissue samples. This extraction protocol has several benefits compared to independent extractions. First, this single extraction saves time and reduces reagent costs (Supplementary Table 2). Most importantly, this approach reduces the amount of material required and can be useful for human biopsies, or whenever a limited amount of tissue is available.

Lipid analysis is often combined with transcriptomic analysis to investigate the etiology of metabolic diseases, such as non-alcoholic liver diseases^12^. Non-alcoholic fatty liver disease (NAFLD) is a major public health concern worldwide^13^. Lipidomics is an important tool in pre-clinical^14,15^ and clinical studies^16–19^ of this continuum of pathologies associated with abnormal storage of hepatic triglycerides^12^. In addition to lipid metabolic profiling, the regulation of gene expression is of major interest in the field because the control of hepatic gene expression by several key transcription factors plays an essential role in NAFLD. LXR is an influential transcription factor in the liver and in NAFLD^20^.

In this study, LXR activity was induced pharmacologically *in vivo*. We were then able to detect a correlation between LXR-dependent changes in hepatic gene expression and lipid profiles using the dual extraction protocol. Consistent with the well-established effect of LXRs on hepatic lipogenesis^4–6^, we found that a significant increase in the expression of lipogenic genes correlated with an increased accumulation of liver triglycerides. Furthermore, in accordance with an associated increase in *Scd1* expression^21^, we also observed an increase in the abundance of hepatic MUFAs.

We also used this experimental setting to question the co-regulation of other genes related to hepatic fatty acid metabolism, with a specific interest in lipid droplet-associated proteins^7,22^. These proteins are likely to influence the etiology of NAFLD, which has been associated with pharmacological^4,6,23^, genetic^24^, and nutritional^25,26^ regulation of LXR-dependent lipogenesis. Interestingly, we found that PPARγ2 and two lipid droplet-associated proteins, namely FITM2 and PLIN3, exhibit similar gene expression pattern as lipogenic enzymes and direct LXR targets. Importantly, other proteins from the PLIN family, FITM1, and the lipid droplet-associated CIDEs, do not share this expression pattern. This finding suggests that marked up-regulation of triglyceride accumulation induced by *de novo* lipogenesis upon pharmacological activation of LXR activity may influence PPARγ2 expression, as well specific subsets of lipid droplet proteins, such as FITM2 and PLIN3.

Finally, we used a condition-specific network analysis^3^ to obtain additional benefits from the dual extraction performed on single samples in these experiments. This analysis highlighted other LXR-dependent modulations of gene expression, such as the up-regulation of *Gpat1* and *Scd2*, which are part of a large group of lipogenic enzymes regulated by LXR. Our results further confirm that TO901317 up-regulated hepatic Scd2 expression^21^ and established that LXR is required in this process. Interestingly, *Dgat2*^8,9^, which encodes the last enzyme in the pathway leading to triglyceride synthesis, appears to be an exception in this pathway, as its pattern of expression reveals an LXR-dependent inhibition in response to the LXR agonist. This inhibition of *Dgat2* may represent an important rate-limiting step regulating a feedback loop in LXR-mediated triacylglycerol synthesis.

The method developed here may be particularly relevant to allow both transcriptome and lipid analysis in high throughput analysis of metabolic diseases, such as NAFLD. The approach may also be very useful for the analysis of biopsies to explore both the transcriptome and lipidome, especially when the amount of tissue is limited, and provide novel opportunities for multi-omic data analysis through sample-specific network analysis.

## Methods

### Animals

LXRα and LXRβ double-knockout mice (LXR^–/–^) and their wild-type controls were maintained on a mixed-strain background (C57BL/6:129 Sv), housed in a temperature-controlled room with a 12-h light/dark cycle, and fed *ad libitum* with water and Global-diet 2016S (Harlan, Gannat, France). Mice of both genotypes received T0901317 (Sigma, Saint-Quentin Fallavier, France, 30 mg/kg of body weight per day) or vehicle (0.5% carboxymethyl-cellulose, 0.5% Tween80) by daily gavage for 4 days^23^. These mice were kindly provided by Dr. D.J. Mangelsdorf (University of Texas Southwestern Medical Center at Dallas, Dallas, TX). All experiments were performed on 19-week-old male mice.

### Ethics approval

All experiments were approved by the relevant Animal Care and Use Committee (CEEA-86, Ministry of Research and Higher Education, France); notification TOXCOM133, and conducted in accordance with the European directive 2010/63/UE.

### Cell culture

JWZ murine hepatic cells, also referred to as MuSH immortalized hepatocytes, were kindly provided by Dr. J.P. Gray^27^. The cells were cultured with L-glutamine (2 mM), penicillin (100 IU/mL), streptomycin (100 mg/mL), and FBS (10%). The culture medium (DMEM, Invitrogen Cergy Pontoise, France) was supplemented with dexamethasone (1 mM).

### RNA extraction

Total RNA was extracted from liver samples or mouse hepatic cells using TRIzol reagent (Invitrogen, Cergy Pontoise, France) according to the manufacturer’s protocol or the standard protocol described by Chomczynski and Sacchi^10^.

### Extraction of lipid from the organic phase

After the addition of chloroform and centrifugation, the aqueous phase was retrieved and used for further RNA extraction. A sample of the organic phase obtained after RNA extraction was used for lipid extraction. We either carefully removed all or 25 µL of the organic phase and added it to a tube containing 2.5 mL of methanol and the appropriate internal standards. The internal standards were: heptadecanoic acid for fatty acid methyl esters, stigmasterol for cholesterol, cholesteryl heptadecanoate for cholesterol esters, and glyceryl triheptadecanoate for triglycerides (all from Sigma). After vortexing, the sample was filtered on glass wool prior to the addition of chloroform (2.5 mL) and water (2 mL). Next, the samples were mixed intensely by vortexing for 10 sec and centrifuged for 2 min at 1500 × g. Finally, the organic phase was extracted, evaporated to dryness, and resuspended in ethyl acetate for neutral lipid analysis or transmethylated for fatty acid analysis.

### Extraction efficiency and estimation of the average organic phase volume

The extraction efficiency was assessed using radiolabeled [1-^14^C] palmitic acid (Perkin Elmer Life Sciences, Paris, France) added prior to the extraction of mRNA from 20 liver samples weighing between 20 and 120 mg. The amount of radioactivity in both phases was then measured. The total organic phase volume could be calculated from the amount of radioactivity found in 25 µL of the organic phase compared to the input measured by liquid scintillation counting (Tri-Carb 1600 TR, Packard, Meriden, CT, USA).

### Standard lipid extraction

Triglycerides, cholesterol, cholesterol esters, and fatty acids were quantified as described previously^14^. Briefly, following homogenization of tissue samples in methanol/5 mM EGTA (2:1, v/v), lipids corresponding to an equivalent of 1 mg of tissue were extracted in chloroform/methanol/water (2.5:2.5:2.1, v/v/v) in the presence of appropriate internal standards according to Bligh and Dyer’s method.

### Neutral lipid analysis

Lipids extracted from the organic phase after RNA extraction or by standard lipid extraction were resuspended in 50 µL of ethyl acetate and neutral lipids analyzed by gas-liquid chromatography on a Focus Thermo Electron system using a Zebron-1 Phenomenex fused-silica capillary column (5 m, 0.32 mm internal diameter, 0.50 mm film thickness). The oven temperature was programmed from 200 to 350 °C at a rate of 5 °C/min, with hydrogen as the carrier gas (0.5 bar). The injector and detector were at 315 and 345 °C, respectively.

### Fatty acid analysis

Fatty acids were analyzed as fatty acid methyl esters (FAMEs) as described previously^28^. The dried lipid extract was transmethylated with 1 mL of BF3 in methanol (1:20, v/v) for 60 min at 80 °C, evaporated to dryness, and the FAMEs extracted with hexane/water (3:1). The organic phase was evaporated to dryness and dissolved in 50 µL ethyl acetate. One microliter of FAMEs was analyzed by gas-liquid chromatography on a 5890 Hewlett-Packard system (Hewlett-Packard, Palo Alto, CA) using a Famewax fused-silica capillary column (30 m, 0.32 mm internal diameter, 0.25 mm film thickness; Restek, Belfast, UK). The oven temperature was programmed from 110 to 220 °C at a rate of 2 °C/min, with hydrogen as the carrier gas (0.5 bar). The injector and detector were at 225 and 245 °C, respectively.

### Real-time qPCR

For real-time qPCR, RNA samples (2 µg) were reverse-transcribed using SuperScript™ II reverse transcriptase (Invitrogen). Primers for SYBR Green assays are given in Supplementary Table 3. Real-time amplification was performed on an ABI Prism 7000 SDS (Applied Biosystems). All qPCR data were normalized to the mRNA levels of TATA box binding protein. Differential gene expression was calculated by the ΔΔC_T_ method.

### Statistical analysis

Data obtained with the different extraction protocols (Fig. 1) were analyzed using R (www.r-project.org). Data are expressed as the mean ± s.e.m. Differential effects were analyzed by ANOVA followed by Student’s t-tests with a pooled variance estimate. A P-value ≤ 0.05 was considered significant. To compare methods, Bland-Altman analyses were performed using GraphPad Prism5 software (GraphPad Software, San Diego, CA). Briefly, we analyzed the ratio of the difference between the results of dual extraction and the reference method (Bligh and Dyer) to the average of the two methods. We determined the bias ± s.d. between dual extraction and the reference method^3^ and verified that the results were included in the 95% limits of agreement (bias ± 1.96 s.d.).

### Single sample network-based analysis

Initially, SSN analysis was developed to analyze transcriptomic data from a single sample compared to a large bank of reference samples^3^. To adapt this method to our set of data, we made some modifications. First, we used the mRNA expression levels of the 74 genes related to lipid metabolism and four sets of data from hepatic lipid analysis (i.e., cholesterol, cholesterol ester, and triglyceride contents and MUFA/(SFA + MUFA) ratio) from LXR^+/+^ and LXR^−/−^ mice treated with or without TO901317 (n = 5 mice per group). Every calculation step was performed with R software (www.r-project.org) and a network graph generated using Cytoscape 3.5.1 software (www.cytoscape.org). After normalization to the mean of the reference groups, we analyzed the distribution of data by performing a Shapiro test. As we excluded our data following a normal distribution, we decided to apply Spearman coefficient of correlation (SCC) instead of the Pearson correlation proposed by Liu *et al*.^8^. We calculated the SCC between each pair of biological parameters in the reference group (SCCref determined on five mice). We then calculated a new SCC as proposed by Liu *et al*.^8^ by adding one mouse to the reference group (SCCref + 1) and determined differences between the two sets of SCCs (ΔSCC1 = SCCref +1− SCCref). We proceeded like this for all of the mice.

SSN analysis was initially developed to compare one specific sample to a large reference group, with important inter-individual variability; however, in our experiment, our reference group was composed of only five mice, with low inter-individual variability due to their common genetic strain, and it was used to make comparisons with three groups of five mice. Therefore, for these five mice per specific group, the median of five individual ΔSCC values was chosen to investigate network perturbations. The choice of the median instead of the classical arithmetic mean was made to avoid overweighting one individual amongst the five mice in a group, thereby improving the significance of the network analysis. Therefore, we finally obtained three sets of median ΔSCC (for TO901317-treated group of LXR^+/+^ mice and TO901317-treated and untreated groups of LXR^−/−^ mice). For each pair of these median ΔSCC values per group, a z-score was calculated and used to determine a P-value for the null hypothesis of equality between this pair of values and the mean of all pairs in this group. Based on this P-value, we selected all ΔSSC pairs including lipid values with P < 0.05. The selection finally showed genes and lipids for which there was a significant change in correlation compared to the reference group (i.e., all gain or loss of correlation in a pair of biological parameters that could be correlated, anti-correlated, or not correlated initially).

## Electronic supplementary material

Supplementary Information
