# Supplementary material for: Dual extraction of mRNA and lipids from a single biological sample
Source: Sci Rep. 2018 May 4;8:7019. doi: 10.1038/s41598-018-25332-9 (PMC5935724; doi:10.1038/s41598-018-25332-9)
Supplement: Supplementary file 1 — Supplementary Information [file 41598_2018_25332_MOESM1_ESM.pdf]

Dual extraction of mRNA and lipids from a single biological sample

Normand Podechard[1,2#], Simon Ducheix[1#], Arnaud Polizzi[1], Frédéric Lasserre[1], Alexandra Montagner[1,3], Vincent Legagneux[2], Edwin Fouché[1], Fabrice Saez[4], Jean-Marc Lobaccaro[4,5], Laila Lakhal[1], Sandrine Ellero-Simatos[1], Pascal. G. Martin[1], Nicolas Loiseau[1], Justine Bertrand-Michel[3,6], Hervé Guillou[1*]

Supplementary Table 1 : Efficiency of radiolabeled [1-^{14}C] palmitic extraction using standard (TRIzol) mRNA extraction

	Dpm in upper phase	Dpm in lower phase	% dpm extracted
**Mean (n=19)**	50.59	10812.85	99.52
**SD (n=19)**	33.29	762.73	0.35

Supplementary Table 2 : Estimated benefit made using dual extraction instead of independent extractions

	Time (min)	Reagent (euros)	Biological material (µg)
Independent extractions	22	35	10
Dual extraction	20	30	5
Benefit / sample	2	5	5

Supplementary Table 3: Oligonucleotide sequences for real time PCR

**Gene**	**NCBI Refseq**	**Forward primer (5'-3')**	**Reverse primer (5'-3')**
Abcg5	NM_031884	TCGCCACGGTCATTTTCA	GCCAAAAGAGCAGCAGAGAAATA
Abcg8	NM_026180	ATCCATTGGCCACCCTTGT	GCGTCTGTCGATGCTGGTC
Acaca	NM_133360	TTACAGGATGGTTTGGCCTTTC	CAAATTCTGCTGGAGAAGCCAC
Acacb	NM_133904	CCTGAATCTCACGCGCCTA	CAGATGGAGTCCAGACATGCTG
Acly	NM_134037	AAAGCTTGGCCTCGTCGG	GGGACGAAGGGTTCAATGAGA
Adhb5	NM_026179	TGGCTTCCTACCTGGTGTCC	GCGCACAGGCTCTTTCTTGTA
Agpat1	NM_018862	CATGAGGCTAGAAGGGAAGACG	CCGGGCCACAGCTCC

Agpat2	NM_026212	CGCACCGTGGATAACATGAG	CTCTGGTGATTAGAGATGATGACACA
Agpat4	NM_026644	ATGCCAGACTCTGCTACTGCG	CAGGCCTTCGGGTCGG
Agpat6	NM_018743	AGCTTTGAAATTGGAGCCACTG	CAATGGCCCAACTGGTCATC
Ccta	NM_009981	TTCCTTCCAAAGTGCAGCG	CCTGACATAGGGCTTACTAAAGTCAA
Cd36	NM_007643	GTTAAACAAAGAGGTCCTTACACATACAG	CAGTGAAGGCTCAAAGATGGC
Cept1	NM_133869	GAAACAGGCCAGAAGAACTAACAGTA	GCAATACAAGTTCCAAGAACCACA
Chka	NM_013490	AGGCCTTCGCGAGGACC	CAACGCTGGCTATGGAGTCTG
Chkb	NM_007692	CTTGGAACAGTACCTCCCAAGC	CCCGACAACACTGGGTCC
Chpt1	NM_001146690	GCTCATCTTCTACTGCCCTACAGTC	GGGCACATAAAAGGTATGTCCAG
Chrebpa	NM_021455	ACTCAGGGAATACACGCCTACAG	GAAGAAGGAATTCAGAGCTCAGAAA
Chrebpb	NM_021455	TCTGCAGATCGCGTGGAG	CTGTCCCGCCATAGCAAC
Cidea	NM_007702	CTACGCGGGAGCCCTCA	GGGCGAGCTGGATGTATGA
Cideb	NM_009894	CCAGCCTCCAAGAACTGCTAGATA	CAGCTGGAAGAAGTCCTCACTGT
Cidec	NM_178373	CATGAAGTCTCTCAGCCTCCTGTA	CAGCTGTTGGGTCACCACTG
Crtc2	NM_028881	ACCCACCTTGTCTTCCATCACT	GGGTGGATGGGTGGGTATAACT
CrebH	NM_145365	CATTGATGGTCTGGAGAACCG	TTGCTGGTTGACTGGACCAC
Cyp3a11	NM_007818	TCACACACACAGTTGTAGGCAGAA	GTTTACGAGTCCCATATCGGTAGAG
Cyp4a10	NM_010011	TCCAGCAGTTCCCATCACCT	TTGCTTCCCCAGAACCATCT
Cyp7a1	NM_007824	AGAGCAACTAAACAACCTGCCAGTA	GCACTGGAGAGCCGCAGA
Dgat1	NM_010046	GGACCAGCGTGGGCG	AAGAATCTTGCAGACGATGGC
Dgat2	NM_026384	ACAGCTGCAGGTCATCTCAGTACTA	AGCACAGCTATCAGCCAGCA
Dhcr24	NM_053272	ATATCTACTACTACGTGCGCGCC	CTGCCCTGTTCCTTCCATTC
Dhcr7	NM_007856	GACCCTCATTAACCTGTCCTTCG	CCAGGTTTCATTCCAGAAGAAGTC
Eci	NM_010023	GTTCACCATCAGCCTGGAGAAG	AGAAGATACCCGGGCATTCC
Elovl1	NM_019422	GTACCTACACCTGGCGCTGTG	CAGGCCACTCGAACCATCC
Elovl2	NM_019423	CAGCTGGGAAGGAGGTTACAACT	AATCGTGTCCAGGAACTCCACTA
Elovl3	NM_007703	CGTAGTCAGATTCTGGTCCT	CCAGAAGAAGTGTTCCGTTG
Elovl4	NM_148941	GCTTAACCTTTTCATCTTCAGAGAGC	CTGACTGGCAAATATAGCTGTATCCT
Elovl5	NM_134255	TCGATGCGTCACTCGTACCTATT	ATTTTGGTCCCAGCCATACAAT
Elovl6	NM_130450	TCTGATGAACAAGCGAGCCA	TGGTCATCAGAATGTACAGCATGT
Fabp	NM_017399	GGCAAGTACCAATTGCAGAGC	AGGTCCTCGGGCAGACCTA
Fads1	NM_146094	TCAACATGCACCCCCTCTTC	GATGGTTGTATGGCATGTGCTT
Fads2	NM_019699	TCCAGTACCAGATCATCATGACAA	GGTGTAGAAGAAACGCATATAGTAGCTG
Fads3	NM_021890	TCATCGGCCACCACGG	TGCGCACAAAATGGAGATCTT
Fads6	NM_178035	TATCTCCATGTCAATATCTTCCAGCA	TGGATCCTCCTGGGCTTCT
Fasn	NM_007988	AGTCAGCTATGAAGCAATTGTGGA	CACCCAGACGCCAGTGTTC
Fdft1	NM_010191	AGGAGTTCTATAACCTGCTGCGAT	GGTCTTCAAGCTGCTGCTGAGT
Fdps	NM_134469	TGCTATTGCCCGGCTCA	ATCCTGTTTCTTCGGCTCCA
Fitm1	NM_026808	CCACGGCAACTTCTTCAACATAA	CACAAAGCCCCCCAGGA
Fitm2	NM_173397	CCGAGAGCTACCTCAGCAACA	AAGGGCAATGAAAGGCAGG
Fxr	NM_009108	CCACCGGCTGTCAGGATT	CGCGTGTTCTGTTAGCATACCTT
G6pc	NM_008061	CTCACTTTCCCCACCAGGTC	GCTGAAAGTTTCAGCCACAGC
Gck	NM_010292	TCGCAGGTGGAGAGCGA	TCGCAGTCGGCGACAGA
Glut2	NM_031197	TTTGCAGTGGGCGGAATGG	GCCAACATTGCTTTGATCCTT
GOs2	NM_008059	TTCCCACTGCACCCTAGGC	TGGCCAGAGGGATCAGCTC
Gpat1	NM_0081449	AGACGAAGCCTTCCGACGA	TGGACATGATAGCGCAGGACT

Hmgcr	NM_008255	CTTGTGGAATGCCTTGTGATTG	GAAGAATGTCATGAACACAAAGTAGTTG
L.Pk	NM_013631	TCGACTCAGAGCCTGTGGC	AGTCGTGCAATGTTCATCCCT
Lipin1	NM_172950	ATGTTTCCCATAGAGATGAGCTCG	GAATGGTGGTACATCATTAGGAAGAG
Lipin2	NM_001164885	AGGACAATAGGAAGGAGGAGCAG	TTGTAGTCCTCTTCCTTTAAGGAAGC
Lipin3	NM_022883	CCCTCTGGGCATCCACAA	TGGCCCCCATCCCATACT
Lpcat3	NM_145130	TACAAGGACAGCTACCTCATCCATC	GAAGCACGACACATAGCAAGGA
Lss	NM_146006	ATGAGTTGGGTCGGCAGAGAT	GCGCTTTTGGTAAGTCCGTG
Lxra	NM_013839	GGAGTGTCGACTTCGCAAATG	TCAAGCGGATCTGTTCTTCTGAC
Me1	NM_008615	CATTCGAGGCGTTTCGTTG	CAGGTAGGATCTGGTCATAATTAGTGC
Mttp	NM_008642	TCAGGAAGCTGTGTCAGAATGAAG	TTTCAAGTCCTCCCAGGATCA
Mvk	NM_023556	GCTTCAGCGACTGGACACG	ACAGGTAGAGAAAGGCAAGCAGA
Pcsk9	NM_153565	AGGAAGACCGCTCCCCTG	TGGTATCTAAGAGATACACCTCCACCT
Pdk4	NM_013743	ATCGCCAGAATTAAACCTCACAC	TGGATTGGTTGGCCTGGA
Pepck	NM_011044	GAACCCCAGCCTGCCC	GAGCAACTCCAAAAAACCCG
Pfk	NM_008826	CTACCGTGGACCTGGAGAAACT	GCCCTGACAGCAGCATTCAT
Plin3	NM_025836	GGCTGGACAGACTGCAGGA	TCTTGAGCCCCAGACACTGTAG
Plin5	NM_025874	CGCTCCATGAGTCAAGCCA	CTCAGCTGCCAGGACTGCTA
Pltp	NM_011125	GGATTAAAGTGTCCAATGTCTCCTG	GTGGAGAAAAAGTTATACATCCTCCTG
Pmdci	NM_016772	GGAAAGATGTTCACTTCAGGTATTGAC	CGGGCCGCATCATCTC
Pmvk	NM_026784	GGAAGGCGTGTCCCAGC	GCCCCATAGGCCTCCTGA
Plin1	NM_175640	GAGAGTAAGGATGTCAATGAACAAGG	GCTTCTTTGGTGCTGTTGTAGGT
Plin2	NM_007408	CCATTTCTCAGCTCCACTCCAC	GTGTCGTCGTAGCCGATGC
Plin3	NM_025836	GGCTGGACAGACTGCAGGA	TCTTGAGCCCCAGACACTGTAG
Plin4	NM_020568	ACCTCCACAAAGGACTTACAAACAG	ACTTCCCATGTCCTTGTCTCCA
Plin5	NM_025874	CGCTCCATGAGTCAAGCCA	CTCAGCTGCCAGGACTGCTA
Pnpla2	NM_001163689	AGTGTCCTTCACCATCCGCTT	GGATATCTTCAGGGACATCAGGC
Pnpla3	NM_054088	ACGCGGTCACCTTCGTGT	AGCCCGTCTCTGATGCACTT
Polr2a	NM_009089	AAGAGAGTGCAGTTCGGAGTCCT	TCCGTTGTTTCTGGGTATTTGA
Ppara	NM_011144	CCCTGTTTGTGGCTGCTATAATTT	GGGAAGAGGAAGGTGTCATCTG
Pparg1	NM_011146	CCACCAACTTCGGAATCAGCT	TTTGTGGATCCGGCAGTTAAGA
Pparg2	NM_011146	ATGGGTGAAACTCTGGGAGATTCT	CTTGGAGCTTCAGGTCATATTTGTA
Scd1	NM_009127	CAGTGCCGCGCATCTCTAT	CTGACTGGCAAATATAGCTGTATCCT
Scd2	NM_009128	CCCCTACGACAAGAACATTAGC	GGTAGTTGTGGAAGCCCTCG
Sec14l1	NM_028777	TCCTTGTCCCAGATGCTGCT	GATGTAGTCGGCATCTAATTTATCGT
Serpinf1	NM_011340	TCAGAGTGCAGGCTGTGAGAGA	CCAGAGGAGTAGCACCAGGG
Shp	NM_011850	CCCAAGGAGTATGCGTACCTGA	TGTGCGATGTGGCAGGAG
Sirt1	NM_019812	GCTGTGAAGTTACTGCAGGAGTGT	CCGCAAGGCGAGCATAGATA
Soat1	NM_009230	TGTTGGCAGCAGAGGCG	GGTCACAAAGTCATCGAAGTGG
Spot14	NM_009381	AACGGAGGAGGCCGAAGAAG	GTTGATGCACCTCGGGGTCT
Sqle	NM_009270	GGAGGCTACCGTGTTCTCCA	CTGCACTTGGTTGGTTTCTGAC
Srebp1c	NM_011480	CAGACACTGGCCGAGATGTG	CTTGGTTGTTGATGAGCTGGAG
Srebp2	NM_033218	GTACTGCGCCCAGAGGAGC	GCCTGAGGTTTCACCAAGGAC
Star1	NM_011485	AAGGCCTTGGGCATACTCAAC	TGGCACCATCTTACTTAGCACTTC
Sult1e1	NM_023135	ATTTCACTTCTTCCACGGGAAC	CCAAAAACTTCATAATACTCAGGCATAG
Tbp	NM_013684	ACTTCGTGCAAGAAATGCTGAA	GCAGTTGTCCGTGGCTCTCT
28S	NR_003279	TGCCATGGTAATCCTGCTCA	CCTCAGCCAAGCACATACACC

Supplementary Figure 1

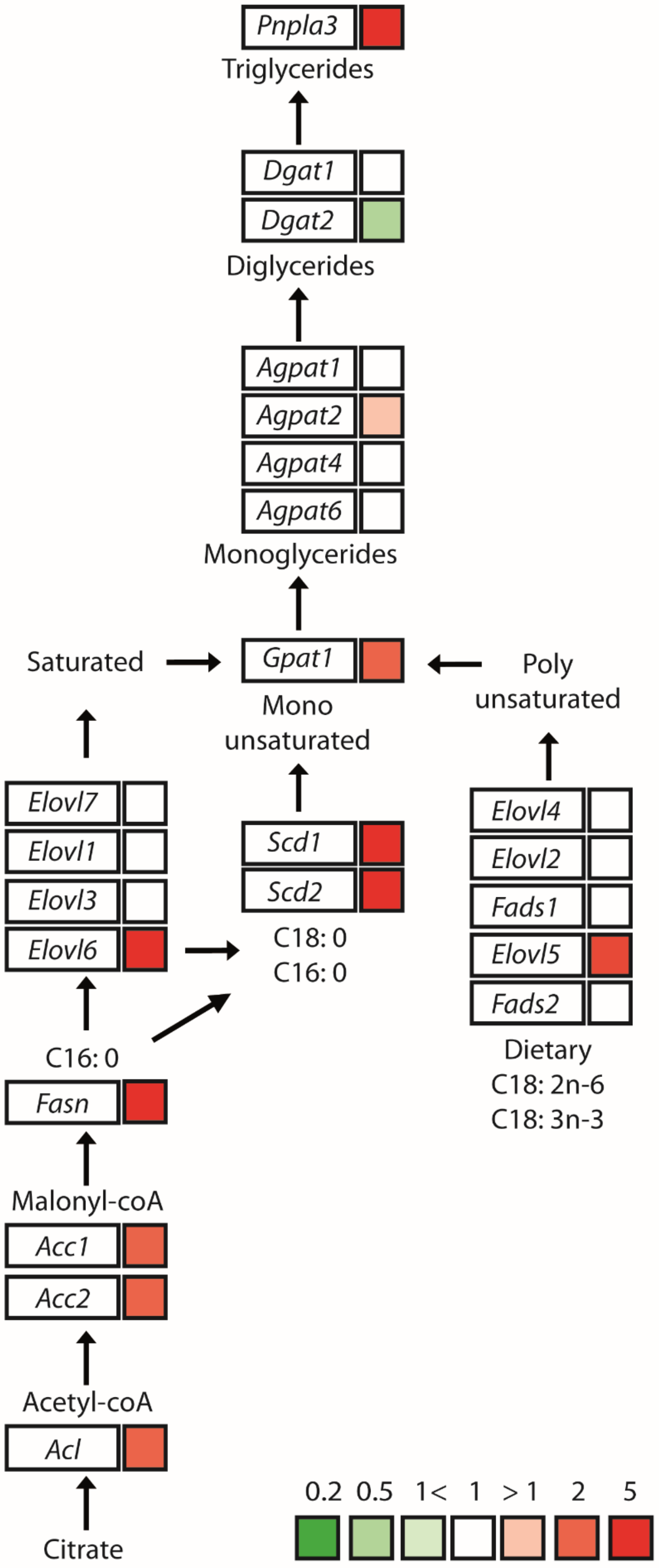

LXR sensitive genes involved in fatty acid and triglyceride synthesis.
Colors relate to the fold-change in mRNA level when a significant effect of T090173 is observed in wild-type but not in knock-out mice lacking LXRs.
